# Tertiary lymphoid structures in chronic rhinosinusitis with nasal polyps: spatial organization, pathological functions, and implications for surgical strategy and biologic therapy

**DOI:** 10.3389/fimmu.2026.1914559

**Published:** 2026-09-01

**Authors:** Yu-Shan Lin, Guan-Jiang Huang, Biao-Qing Lu, Qi-Ping Luo, Zhi-Jun Fan, Xue-Sen Yang, Chao-Qing Long

**Affiliations:** 1 Department of Otolaryngology, Zhongshan Hospital of Traditional Chinese Medicine, Zhongshan, China; 2 The Tenth Clinical Medical College of Guangzhou University of Traditional Chinese Medicine, Zhongshan, China

**Keywords:** chronic rhinosinusitis with nasal polyps, CXCL13, dupilumab, functional endoscopic sinus surgery, germinal center, spatial transcriptomics, tertiary lymphoid structures, tissue-resident memory T cells

## Abstract

Chronic rhinosinusitis with nasal polyps (CRSwNP) presents three persistent clinical enigmas that conventional immunological frameworks have failed to resolve. These include the paradox of locally elevated specific IgE in the absence of systemic sensitization, a high polyp recurrence rate exceeding 60% following technically complete functional endoscopic sinus surgery (FESS), and disease rebound observed in the majority of patients within 6 to 12 months after discontinuation of dupilumab. Converging lines of evidence suggest that tertiary lymphoid structures (TLS), organized ectopic lymphoid aggregates capable of sustaining autonomous adaptive immune responses, may represent a unifying structural framework that could contribute to all three phenomena. Within CRSwNP tissue, mature TLS are proposed to harbor functional germinal centers that drive local IgE class-switch recombination independent of systemic sensitization. These structures may also serve as protected niches for tissue-resident memory T (TRM) cells refractory to systemic biologic blockade, and they may maintain self-sustaining immune circuits that persist after surgical removal of gross polyp tissue. This review synthesizes current knowledge of TLS formation biology, proposes a Trifunctional TLS Model as a conceptual framework. It further integrates recent spatial transcriptomic data from large cohorts to hypothesize that TLS anatomical depth may determine functional phenotype and therapeutic vulnerability. We further propose a TLS grading framework as a research instrument to structure future validation studies, and outline hypothesis-driven, testable implications for surgical extent, biologic targeting strategies, and future research directions. If validated, reframing CRSwNP as a TLS-driven, structurally semi-autonomous inflammatory disease could shift the therapeutic paradigm from symptom suppression toward immunological restructuring.

## 1 Introduction: three unanswered clinical questions

CRSwNP is a chronic, type 2 inflammation-dominated disease of the sinonasal mucosa affecting 0.5% to 4.0% of the general population, characterized by bilateral nasal polyposis, impaired olfaction, and a profound burden on quality of life (1). Over the past decade, biological therapies targeting key type 2 cytokines, including dupilumab (anti-IL-4Rα), mepolizumab (anti-IL-5), and omalizumab (anti-IgE), have dramatically expanded treatment options for patients with severe, recalcitrant disease (2–5). Nevertheless, three fundamental clinical paradoxes remain unresolved by prevailing models of CRSwNP immunopathogenesis. These three paradoxes, immunological, surgical, and therapeutic, serve as the organizing framework of this review. We return to each of them in turn when evaluating the explanatory scope and limits of the proposed model.

The first paradox is immunological. A substantial proportion of patients with CRSwNP demonstrate markedly elevated local specific IgE in nasal lavage fluid and polyp tissue despite normal serum total IgE, negative skin prick tests, and absence of systemic atopy (6, 7). Classical allergological frameworks posit a unidirectional pathway from systemic sensitization to local effector responses, yet these patients defy this paradigm entirely. Local immunoglobulin class-switching within the nasal mucosa, a process requiring germinal center reactions, somatic hypermutation, and T follicular helper (Tfh) cell collaboration, cannot be explained by circulating IgE redistribution (8). The molecular substrate for this autonomous local humoral immunity demands a structural explanation.

The second paradox is surgical. FESS represents the primary interventional standard for medically refractory CRSwNP, with mounting evidence supporting expanded surgical approaches for severe phenotypes (9). Yet multicenter datasets consistently demonstrate 5-year polyp recurrence rates exceeding 60% in patients with severe eosinophilic CRSwNP, even when polyps have been endoscopically eradicated (10, 11). Conventional explanations invoke persistent mucosal inflammation driven by the cytokine microenvironment. Yet neither intranasal corticosteroids nor biologic maintenance therapy reliably prevents recurrence in high-risk patients after surgery (12). A structural reservoir of immune activity capable of driving local antigen presentation, cytokine production, and eosinophil recruitment independent of systemic inflammatory signals provides a far more parsimonious explanation for the recurrence phenomenon.

The third paradox is therapeutic. Dupilumab has demonstrated exceptional efficacy in reducing nasal polyp score (NPS) and improving sinonasal symptoms in pivotal trials and real-world cohorts, with sustained responses observed over 3 years of continuous therapy (3, 13). However, the drug does not appear to achieve true disease modification. Upon discontinuation, the majority of patients experience significant polyp regrowth within 6 to 12 months, reflecting the reactivation of persistent local immune programs rather than simple cytokine rebound (3, 14, 15). This pattern of effective suppression without resolution is biologically distinct from true remission and suggests that the cellular and structural underpinnings of disease chronicity are not addressed by targeting circulating cytokine signals.

These three paradoxes collectively point toward an underexplored structural mechanism. Under conditions of chronic mucosal inflammation, the nasal mucosa develops highly organized, functionally autonomous lymphoid aggregates, termed tertiary lymphoid structures (TLS), which recapitulate the antigen presentation, lymphocyte activation, affinity maturation, and memory generation functions of secondary lymphoid organs (SLO), entirely within the nasal polyp itself (16, 17). TLS in CRSwNP may function as local IgE factories, protected sanctuaries for tissue-resident memory cells, and autonomous immune control units that could be resistant to both surgical extirpation and systemic pharmacological blockade. The purpose of this review is to consolidate the emerging evidence for TLS as a candidate structural contributor to CRSwNP pathophysiology and to propose the Trifunctional TLS Model as a conceptual framework. We further translate this framework into testable hypotheses for surgical decision-making and the rational design of future biologic strategies.

## 2 TLS biology: from universal concepts to CRSwNP-specific formation mechanisms

### 2.1 The universal biology of TLS

TLS are defined as organized lymphoid aggregates that arise in non-lymphoid tissues under conditions of sustained antigen exposure or chronic inflammation, structurally and functionally recapitulating SLOs in an ectopic setting (16, 17). Unlike transient inflammatory infiltrates, mature TLS possess a compartmentalized architecture comprising segregated T cell and B cell zones, a network of follicular dendritic cells (FDC), and high endothelial venules (HEV) expressing peripheral node addressin (PNAd). This architecture culminates in a functional germinal center (GC) capable of supporting somatic hypermutation, affinity maturation, and class-switch recombination (18).

TLS formation is initiated by a cascade of molecular events orchestrated by lymphoid tissue inducer (LTi) cells. Lymphotoxin-α1β2 (LTα1β2) expressed on LTi cells ligates lymphotoxin-β receptor (LTβR) on local stromal cells, triggering upregulation of the lymphocyte-attractant chemokines CXCL13, CCL19, and CCL21 (17, 19). The CXCL13-CXCR5 axis guides B cells and Tfh cells into nascent follicular zones, whereas CCL19/CCL21 signaling through CCR7 stabilizes the T cell compartment and recruits conventional dendritic cells (16, 20). HEV neogenesis, driven by vascular endothelial activation downstream of LTβR signaling, enables continuous recruitment of naïve and central memory lymphocytes from peripheral circulation (16). The presence of FDC networks, identified by CD21 and CD23 expression, marks the transition to mature TLS capable of sustaining GC reactions with full antibody diversification capacity (18).

In the oncology literature, where TLS research is most advanced, mature TLS correlate strongly with favorable prognosis and enhanced responses to immune checkpoint blockade across multiple solid tumor types (21, 22). Spatial transcriptomic analyses of nasopharyngeal carcinoma have identified CXCL13^+^ cancer-associated fibroblasts, stem-like CXCL13^+^CD8^+^ T cells, and GC-B cell populations as core TLS constituents (20). However, the prognostic and mechanistic implications of TLS in CRSwNP, a non-malignant chronic inflammatory disease, are fundamentally distinct and deserve independent examination (23).

### 2.2 CRSwNP-specific mechanisms of TLS induction

Three interconnected dimensions of CRSwNP biology converge to create an exceptionally fertile milieu for TLS neogenesis.

#### 2.2.1 Microbial dimension

*Staphylococcus aureus* (*S. aureus*) colonizes the nasal mucosa of approximately two-thirds of patients with CRSwNP, a prevalence substantially exceeding that in healthy controls or patients with CRS without nasal polyps (24, 25). The organism releases a family of protein superantigens, most prominently staphylococcal enterotoxin B (SEB). These superantigens engage MHC class II molecules outside the conventional antigen-binding groove, triggering non-specific polyclonal activation of large T cell populations (25, 26). This extensive, sustained T cell stimulation generates the LTα-rich lymphoid tissue inducer environment required for TLS neogenesis, without dependence on cognate antigen recognition (27). Notably, local inflammatory signals exacerbated by *S. aureus* colonization, such as IL-17A, stimulate nasal stromal cells to upregulate CXCL13 expression, establishing a chemokine gradient that specifies the spatial coordinates of incipient TLS (28). Follicle-like structures with local IgE production have been observed in nasal polyp tissue in direct association with *S. aureus* enterotoxin deposits, providing some of the earliest histological evidence for ectopic lymphoid organogenesis in this disease (24, 29).

#### 2.2.2 Epithelial alarm signaling dimension

The dysfunctional nasal epithelial barrier in CRSwNP constitutively releases alarmin cytokines such as thymic stromal lymphopoietin (TSLP), IL-33, and IL-25, which drive multiple downstream inflammatory cascades (12, 30). Beyond their well-characterized role in activating ILC2s and promoting Th2 polarization, these alarmins stimulate local innate immune populations, including mast cells and ILC2s, to produce TNF and LTα, both of which are critical upstream signals for stromal cell reprogramming toward an LTo (lymphoid tissue organizer) phenotype and for HEV neogenesis (17). Single-cell RNA sequencing and spatial transcriptomics of CRS tissue have now demonstrated that immune-epithelial interaction modules, which are characterized by dysregulated basal cell trajectories, macrophage-eosinophil recruitment signals, and CD4^+^ T cell activation programs, are spatially restricted to specific mucosal regions, consistent with focal TLS formation rather than diffuse lymphocytic infiltration (31, 32). The epithelial barrier defect in CRSwNP therefore serves a dual pathogenic role, initiating Th2 polarization while simultaneously providing the structural and biochemical scaffolding for TLS formation.

#### 2.2.3 Temporal dimension

**Figure 1:**
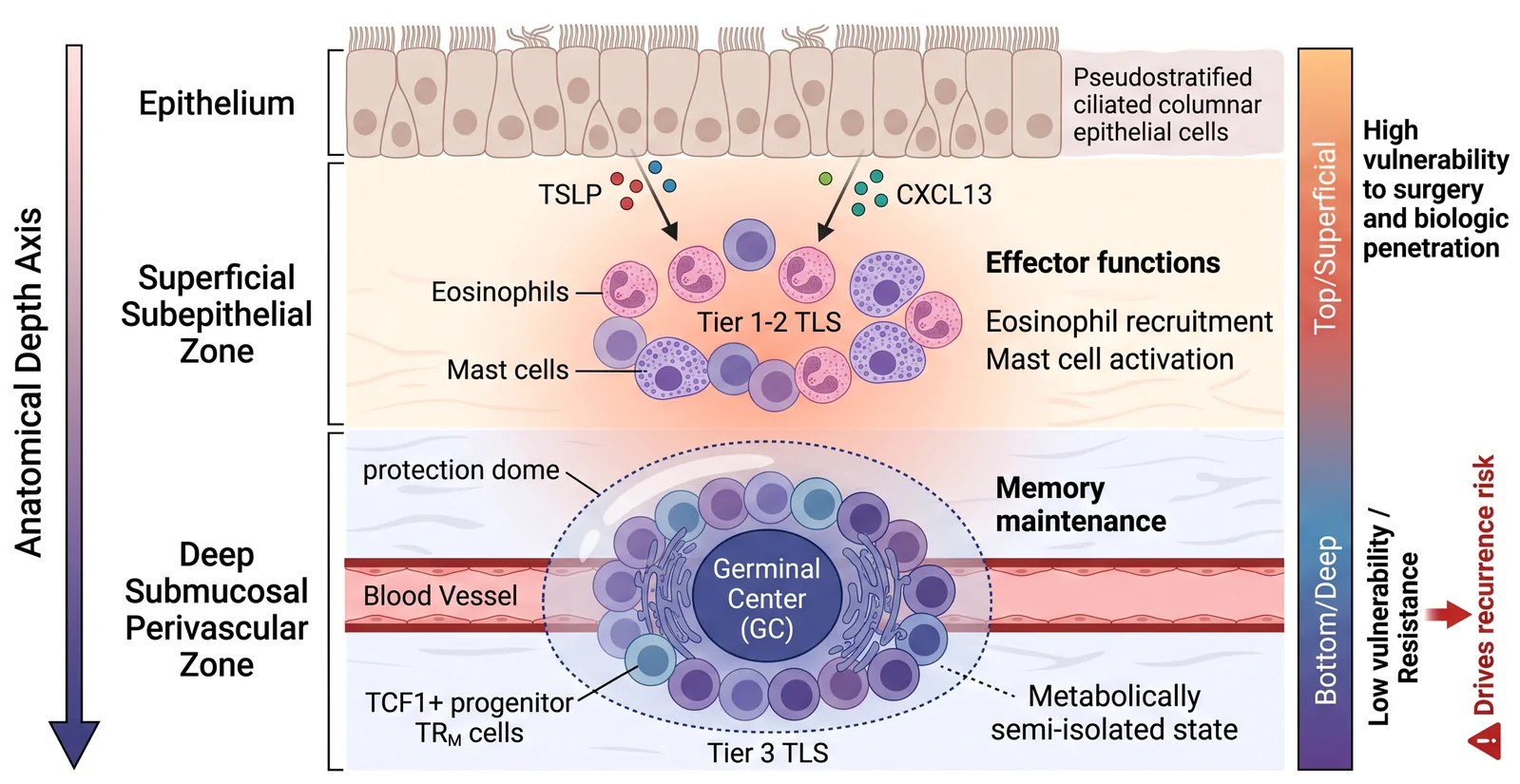
Schematic diagram illustrating the three-tier TLS maturation model in CRSwNP nasal mucosa. Tier 1 represents early-stage diffuse lymphoid aggregates lacking internal zoning. Tier 2 represents immature TLS with nascent T/B cell compartmentalization and early HEV formation. Tier 3 represents mature GC-containing TLS with FDC networks, active germinal center reactions, and CXCL13 gradient-driven B cell and Tfh cell recruitment. Key molecular drivers including LTα1β2, CXCL13-CXCR5, and CCL19/CCL21-CCR7 axes are annotated. Created with BioRender.

CRSwNP is defined by chronicity. Patients present with a mean disease duration exceeding 5 years, and many have undergone one or more prior sinus surgeries before receiving advanced therapies (1, 3). This extended inflammatory timeline provides the temporal window necessary for TLS evolution from loose lymphocytic aggregates (Tier 1) through progressively compartmentalized structures (Tier 2) to fully mature GC-containing TLS (Tier 3) capable of autonomous humoral immunity (**Figure 1**, **Table 1**) (33, 34). This temporal dynamic distinguishes CRSwNP from both acute mucosal infections (which resolve before TLS can mature) and rapidly progressing malignancies (where TLS form quickly but under distinct oncological signaling environments) (16, 35).

**Table 1:** TLS maturation staging system for CRSwNP.

Stage	Tier	Histological features	Immunohistochemical markers	Functional capacity	Surgical vulnerability	Biologic sensitivity
Early lymphoid aggregate	Tier 1	Diffuse T/B cell infiltrate, no internal zoning	CD3^+^, CD20^+^, CXCL13^–^	Limited antigen presentation, effector cytokine release	High (superficial lamina propria)	Moderate–High
Immature TLS	Tier 2	Nascent T/B cell compartmentalization, early HEV	CD3^+^, CD20^+^, PNAd^+^, CXCL13+low	Partial GC reactions, early local IgE switching	Moderate (mid-submucosal)	Moderate
Mature TLS (GC^+^)	Tier 3	Segregated T/B zones, FDC network, active GC	Bcl-6^+^, CD21^+^, CXCL13^+^high, PNAd^+^	Full somatic hypermutation, IgE/IgG CSR, TRM maintenance	Low (deep periosteal)	Low (Sanctuary Effect)

HEV, high endothelial venule; GC, germinal center; FDC, follicular dendritic cell; CSR, class-switch recombination; PNAd, peripheral node addressin; TRM, tissue-resident memory T cell.

The “Functional Capacity,” “Surgical Vulnerability,” and “Biologic Sensitivity” columns represent proposed, hypothesis-generating attributions inferred from TLS maturation biology and cross-disease data. They have not been directly validated in CRSwNP.

## 3 The trifunctional TLS model: a unified pathophysiological framework

**Figure 2:**
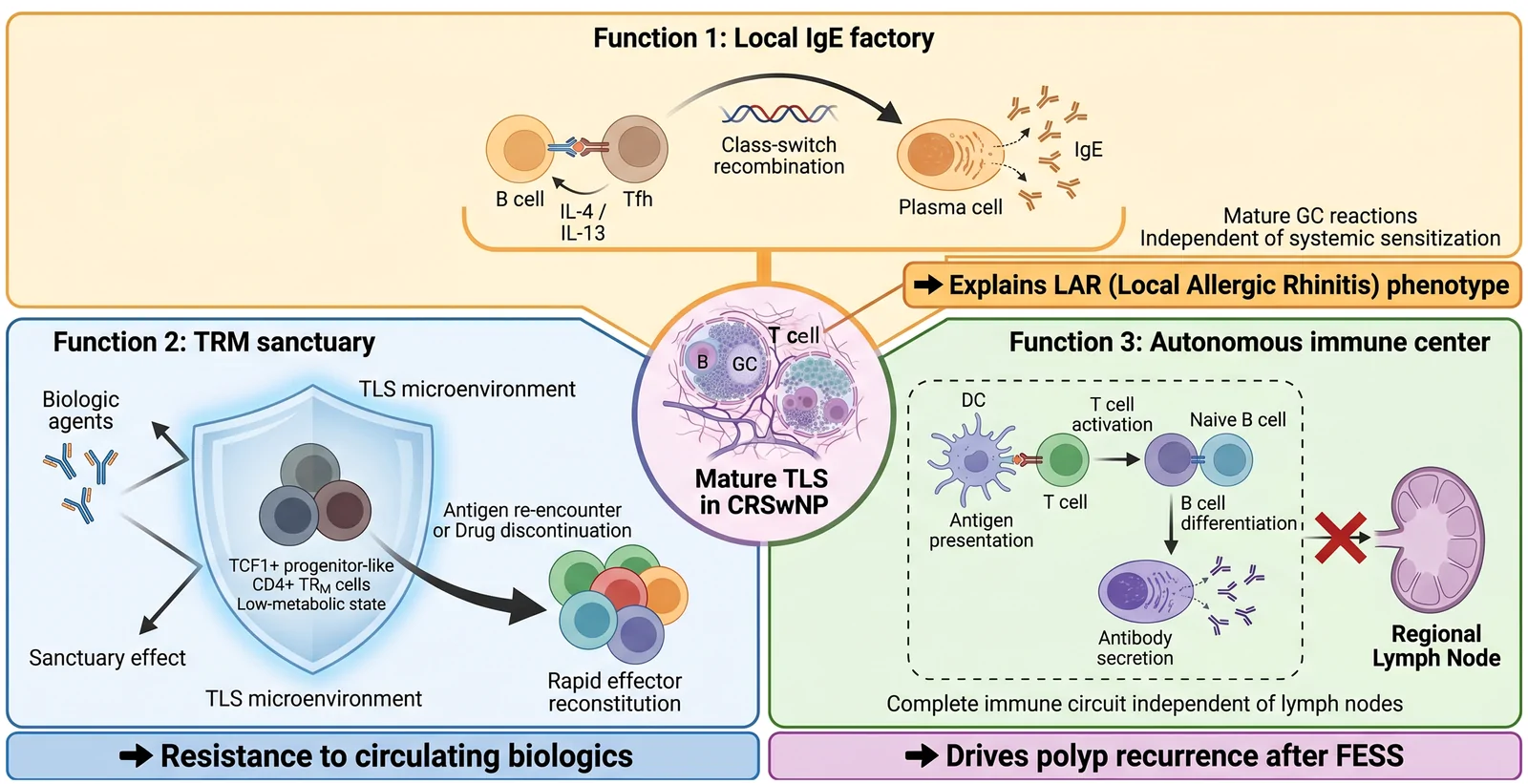
The trifunctional TLS model in CRSwNP (proposed conceptual framework). The three interconnected functions of mature TLS depicted here are hypothesized rather than established in CRSwNP. First, the local IgE factory function reflects mature GC reactions driven by IL-4/IL-13 support B cell class-switch recombination to IgE independent of systemic sensitization, explaining the local allergic rhinitis (LAR) phenotype. Second, the TRM sanctuary function reflects TCF1+ progenitor-like CD4+ TRM cells that reside in low-metabolic states within TLS microenvironments, remain resistant to circulating biologic agents but capable of rapid effector reconstitution upon antigen re-encounter or drug discontinuation. Third, the autonomous immune center function reflects a complete circuit spanning antigen presentation, T cell activation, B cell differentiation, and antibody secretion within TLS that operates independently of regional lymph nodes, driving polyp recurrence after FESS. The sanctuary effect of TLS against biologic therapy is indicated.

We propose the Trifunctional TLS Model to integrate the three cardinal pathological functions of TLS in CRSwNP into a single mechanistic framework (**Figure 2**). This model is proposed to account for the three clinical paradoxes articulated in the Introduction and offers a candidate structural immunological basis for observed treatment failures.

### 3.1 Function I: local IgE factory for resolving the local allergic phenotype

Mature GCs within CRSwNP TLS provide the cellular machinery for B cell clonal expansion, somatic hypermutation, and immunoglobulin class-switch recombination (CSR) (18, 26). The type 2 cytokine milieu of nasal polyp tissue, which is dominated by IL-4 and IL-13 produced by Th2 cells, ILC2s, and mast cells, selectively directs B cell CSR toward IgE (30, 36). This process is thought to be largely confined to the mucosal TLS and to operate independently of systemic sensitization (37).

High-throughput B cell receptor sequencing studies have revealed that IgE-producing plasmablasts within nasal polyps exhibit extensive somatic hypermutation, indicating local affinity maturation rather than migration of pre-formed IgE^+^ cells from systemic circulation (38). Approximately 50% of nasal polyp tissue homogenates from patients with CRSwNP contain IgE antibodies directed against *S. aureus* enterotoxins, supporting a role for the microbiome-TLS axis in local humoral immunity (24). The clinical entity of local allergic rhinitis (LAR), characterized by nasal symptoms and elevated local specific IgE in the absence of systemic atopy, therefore finds its structural explanation in TLS-driven local IgE production, entirely decoupled from serum IgE levels (7, 8).

Within the same TLS framework, preliminary evidence raises the possibility of local autoantibody production directed at self-antigens such as PLEC and DLAT (26, 31). These observations derive from limited cohorts and remain to be independently validated. They should therefore be regarded as hypothesis-generating rather than as an established autoimmune component of the disease, and warrant further investigation.

### 3.2 Function II: TRM sanctuary accounting for drug discontinuation rebound

Recent advances in tissue-resident memory T cell biology have identified a specialized TCF1^+^ progenitor-like CD4^+^ TRM subset that persists within organized lymphoid microenvironments in a quiescent, low-metabolic state (39, 40). Studies of nasal polyp tissue have demonstrated enrichment of CD69^+^CD103^–^CD4^+^ TRM cells, a population showing a pronounced Th2 phenotype that positively correlated with disease severity in eosinophilic CRSwNP (41). The TLS microenvironment, which is sheltered from peripheral blood signals by stromal cell barriers, metabolic gradients, and spatially restricted cytokine niches, provides an ideal ecological niche for TRM maintenance (39, 42).

The critical pharmacological implication, which remains to be tested in CRSwNP, is that TCF1^+^ progenitor-like TRM cells within TLS may be relatively insensitive to systemic biologic agents such as dupilumab (15). In their quiescent, low-metabolic state, these progenitor cells do not rely on IL-4 signaling for *in vivo* maintenance, but rather depend on alternative niche signals such as TSLP (15). Furthermore, systemic monoclonal antibodies distributed through peripheral circulation face significant penetration barriers to access the central zones of mature TLS, where FDC networks and dense stromal cell scaffolding restrict solute diffusion (35). Upon drug discontinuation or antigen re-encounter, these TLS-resident progenitor T cells rapidly differentiate into effector Th2 and Tfh cells, reconstituting the local inflammatory cascade and driving polyp regrowth within weeks to months (43–45).

The following account of restricted biologic penetration is extrapolated from renal cell carcinoma and lymphoma pharmacokinetics (17, 35, 46). No direct measurement of biologic distribution within CRSwNP TLS currently exists, and the Sanctuary Effect is therefore advanced as an analogy-based hypothesis rather than a demonstrated mechanism in this disease. We propose the concept of the TLS Sanctuary Effect to describe this pharmacological refuge, in which TLS in CRSwNP serve as protected microenvironments where the cellular memory of disease is maintained beyond the reach of systemic biologic therapy, explaining the near-universal pattern of disease rebound following drug discontinuation (35, 37). This concept has direct implications for the design of future treatment strategies and offers one mechanistically coherent hypothesis for the failure of current biologics to achieve durable disease modification, although it remains to be tested directly in CRSwNP.

### 3.3 Function III: autonomous immune center explaining surgical failure

The anatomical and functional autonomy of mature TLS from regional lymph nodes and systemic immune surveillance constitutes the third and perhaps most surgically relevant arm of the Trifunctional Model. Mature TLS harbor a complete functional circuit comprising antigen uptake, MHC class II-mediated presentation, T cell priming, B cell activation, GC reactions, and antibody secretion, all contained within a compact mucosal structure measuring hundreds of micrometers to a few millimeters in diameter (17, 46).

Standard FESS achieves high rates of endoscopic polyp clearance. However, its plane of dissection is defined by macroscopically visible disease, and residual submucosa, periosteum, and deep mucosal layers containing Tier 2 and Tier 3 TLS are routinely left in situ (10, 31). These residual TLS, particularly those located in the periosteal and submucosal planes of the ethmoid labyrinth, may continue to produce local IgE, generate CXCL13 and CCL21 gradients that recruit circulating lymphocytes, and activate tissue-resident eosinophils and mast cells (19, 47). Because the TLS-resident immune program is proposed to operate independently of regional lymph drainage, it would not be expected to be disrupted by systemic immune tolerance induction, corticosteroid treatment, or peripheral cytokine blockade (17, 31).

This model provides a candidate structural-immunological rationale for radical mucosectomy with periosteal exposure, the so-called Reboot surgical approach, which aims to physically eliminate mature submucosal TLS together with the inflammatory mucosa, permitting repopulation by a naïve mucosal immune environment (48, 49). Prospective data on the immunological consequences of Reboot surgery remain limited. Nonetheless, the Autonomous Center concept generates a specific, testable prediction: that depth of resection, rather than macroscopic polyp clearance alone, may be the critical determinant of long-term recurrence prevention. This prediction requires direct validation before it can inform surgical practice.

### 3.4 Cellular constituents of CRSwNP TLS: which cells may drive each function

The preceding sections describe TLS as a structure. However, the functional output of a TLS is determined by its cellular composition. Here we dissect the leukocyte and stromal populations that plausibly underlie each arm of the proposed Trifunctional Model, while emphasizing that direct single-cell characterization of bona fide TLS in CRSwNP, as opposed to bulk polyp tissue, remains limited.

CD8^+^ T cells and the autonomous immune center. Recent spatial and single-cell work has identified a GZMK-expressing CD8^+^ T cell population that promotes recurrent airway inflammatory disease (47). Within the Trifunctional framework, GZMK^+^ CD8^+^ T cells are attractive candidate effectors of the “autonomous immune center.” They occupy spatially defined niches in nasal polyp tissue, associate with fibroblast-derived neutrophil chemoattractant programs (50), and could contribute to the self-sustaining, lymph node-independent inflammatory circuit that persists after macroscopic polyp clearance (47). Whether these cells localize to the follicular/peri-follicular compartments of mature TLS, and whether they conform to the proposed superficial-effector versus deep-memory dichotomy (Section 4.2), has not yet been established and represents a specific, testable question for spatially resolved multi-omics.

B cell heterogeneity and the local IgE factory. The statement that “mature TLS harbor functional germinal centers” encompasses several functionally distinct B cell states that should not be treated as a single compartment. Beyond canonical GC-B cells and IgE-switched plasmablasts, which exhibit extensive somatic hypermutation consistent with *in situ* affinity maturation (38). The chronic, TLR- and cytokine-rich milieu of CRSwNP is precisely the setting in which atypical/age-associated B cells (ABCs) arise. ABCs are transcriptionally distinct antigen-experienced B cells that expand in infection and autoimmune disease and are variously defined as CD19^+^, CD21-low, CD11c^+^, T-bet^+^ cells (51, 52). These cells can rapidly differentiate into antibody-secreting cells, secrete inflammatory cytokines, act as potent antigen-presenting cells, and have been hypothesized to drive germinal-center formation and autoantibody production. Because ABCs are induced in part by IL-4 and are enriched for autoreactive specificities, they constitute a mechanistically plausible, though in CRSwNP entirely unproven, source of the preliminary local autoantibody reactivities (e.g., against PLEC and DLAT) (31) noted in Section 3.1. Formal identification of GC-B, IgE^+^ plasmablast, memory, and ABC subsets within CRSwNP TLS, and attribution of local IgE versus autoantibody output to specific subsets, is a priority.

Tfh, Th2, and the Treg/Tfr checkpoint. GC output depends on the balance between T follicular helper (Tfh) support and T follicular regulatory (Tfr) restraint. Of particular relevance to a type 2 disease, a specialized IL-13-producing Tfh subset (“Tfh13”) has been shown to drive high-affinity, anaphylactic IgE (53). Such cells provide a direct cellular bridge between the Th2 cytokine milieu of the polyp and the class-switching machinery of the TLS GC, potentially explaining why local IgE production is so tightly coupled to type 2 inflammation. Countering this, Tfr cells normally constrain the GC reaction. Tfr cells negatively regulate GC responses to prevent autoimmunity but may also support ongoing responses, and can derive from both Treg and Tfh lineages (54). A relative deficiency or functional skewing of local Tfr control offers a coherent mechanism by which autonomous, chronically active GCs could persist in CRSwNP TLS. Conversely, an intact Tfr checkpoint might explain why some polyps never progress to mature GC-containing (Tier 3) structures. This Tfh13-driven amplification/Tfr-restraint axis conceptually links Function I (local IgE) with the durability of Function III (autonomous center), and its dysregulation is a plausible unifying node warranting direct investigation.

Innate and stromal participants as ongoing components, not merely inducers. ILC2s, mast cells, follicular dendritic cells (FDC), and the lymphoid-tissue-organizer (LTo)-reprogrammed stromal compartment are introduced in Section 2.2 as upstream inducers, but they also plausibly function as persistent constituents of the mature structure. FDC networks and dense stromal scaffolding are the very elements invoked for the Sanctuary Effect (Section 3.2). The same stromal biology that restricts solute diffusion also sustains GC organization and CXCL13 gradients, making the stromal compartment simultaneously a structural, functional, and pharmacological determinant of TLS behavior. Notably, mucosal ILC2s can acquire memory-like properties, and allergen-experienced ILC2s have been reported to persist and enhance subsequent allergic inflammation (55). Furthermore, this inflammatory memory is not restricted to immune cells. Respiratory epithelial progenitor cells (basal cells) can also retain intrinsic epigenetic memories of type 2 cytokines (IL-4/IL-13), fundamentally altering the local tissue ecosystem (56). Together, these findings suggest that innate and tissue-level memory embedded within or adjacent to TLS may cooperate with adaptive TRM to reconstitute inflammation after therapy, a possibility that further blurs the boundary between the TLS-centric model and the alternative mechanisms considered in Section 3.5.

### 3.5 Disease heterogeneity and the limits of a TLS-centric model

A framework that positions TLS as the “keystone” of CRSwNP must be tested against the disease’s well-documented heterogeneity and against competing explanations for the same clinical observations. Overreliance on a single mechanism (a “one key for all locks” narrative) is itself a scientific warning sign, and we therefore state explicitly the boundaries of the Trifunctional Model.

#### 3.5.1 Endotype and geographic heterogeneity

Not all polyps are TLS-rich. The model is most applicable to severe, type 2-high, eosinophilic CRSwNP, in which sustained type 2 signaling and *S. aureus*-driven superantigenic stimulation create a permissive milieu for ectopic lymphoid organogenesis. This endotype is not universal. In Western populations, CRS is predominantly type 2 eosinophilic, whereas Asian cohorts show substantially higher rates of non-type 2 endotypes (57), and the traditional one-size-fits-all model is therefore increasingly regarded as invalid across regions (58). In neutrophilic or mixed, IL-17/IFN-γ-skewed disease, which represents a large fraction of Asian CRSwNP, mature GC-containing TLS may be sparse or absent, and mechanisms such as IL-1β-induced epithelial and fibroblast transdifferentiation promoting neutrophil recruitment may dominate (59). Any future TLS grading or TLS-guided treatment strategy must therefore be endotype-stratified, and a “TLS Low” result should be interpreted as biologically informative rather than as a failure of sampling.

#### 3.5.2 Competing and complementary drivers of chronicity

Several established mechanisms can account for local IgE persistence, recurrence, and biologic rebound without invoking TLS, and the accurate position is that these operate alongside, not merely downstream of, any TLS contribution. Foremost is epithelial-intrinsic memory. Epithelial stem cells may contribute to disease persistence by serving as repositories of allergic memory (56), with ex vivo basal cells retaining an intrinsic memory of prior IL-4/IL-13 exposure (56). Because this basal-cell reprogramming is only partially reversed by IL-4Rα blockade, it provides a TLS-independent explanation for post-dupilumab rebound that is at least as parsimonious as the Sanctuary Effect. Similarly, ILC2 tissue memory (55) and aberrant basal-progenitor differentiation trajectories identified by single-cell studies (56) can sustain type 2 inflammation independently of organized lymphoid structures. Rather than subsuming these processes into the TLS narrative, we propose that they are best viewed as parallel, potentially synergistic layers of “tissue memory” (epithelial, innate, and adaptive), of which TLS represents the adaptive, structurally organized tier.

#### 3.5.3 Directionality and causation remain unestablished

Even where mature TLS are present and correlate with severity, the available human data are cross-sectional and associative. TLS may be a driver of chronicity, a bystander marker of long-standing inflammation, or both. The temporal and causal relationships proposed throughout this review are inferred from cross-disease analogy and require the longitudinal and interventional designs outlined in Section 6 before they can be regarded as established. We therefore present the Trifunctional Model as a hypothesis-generating synthesis whose principal value is to structure falsifiable experiments, not as a settled account of CRSwNP pathogenesis.

## 4 Spatial transcriptomics and TLS heterogeneity: position determines function

### 4.1 Spatial mapping of immune-epithelial interactions in CRSwNP

The introduction of spatial transcriptomics, notably 10x Genomics Visium and the recently available Visium HD platform with near-single-cell resolution, has substantially advanced our understanding of the spatial organization of immune responses within complex tissues (31, 60). A large multicenter transcriptomic study published in *Immunity* in 2025 applied both scRNA-seq and spatial transcriptomics to a cohort encompassing over 100 individuals with CRS. This study revealed that immune-epithelial interaction (IMM-EPI) modules are spatially restricted within the nasal mucosa rather than uniformly distributed (32). This spatial clustering of dysregulated immune-epithelial crosstalk, featuring aberrant basal cell trajectories, macrophage activation states, and CD4+ T cell programs, is consistent with a TLS-anchored model of focal immune amplification rather than diffuse mucosal inflammation.

**Figure 3:**
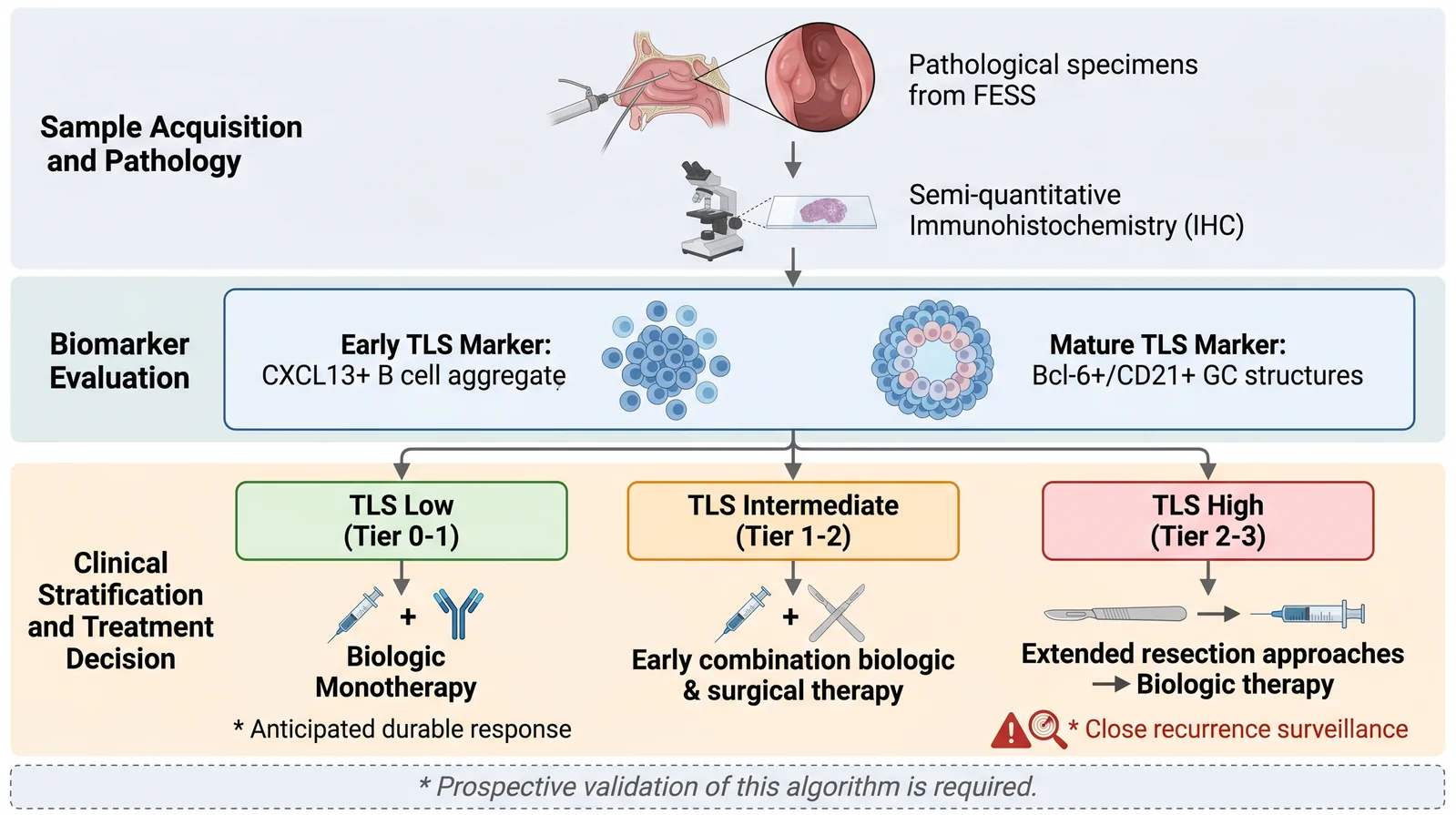
Schematic representation of the proposed spatial heterogeneity of TLS in CRSwNP nasal polyp tissue, informed by, but not yet directly demonstrated by, current spatial transcriptomics data. Superficial subepithelial TLS (Tier 1-2) are closely coupled to TSLP/CXCL13 signals from the epithelium and predominantly execute effector functions including eosinophil recruitment and mast cell activation. Deep submucosal perivascular TLS (Tier 3) harbor GC reactions and TCF1+ progenitor TRM cells, are metabolically semi-isolated from peripheral signals, and preferentially sustain memory maintenance functions. Under this working hypothesis, anatomical depth is proposed to predict differential vulnerability to surgery, biologic penetration, and recurrence risk. This depth-function relationship remains to be validated by single-structure co-registration of TLS maturation stage with mucosal depth.

Parallel spatial transcriptomic studies using the Visium HD platform in nasal polyp tissue have identified discrete regions of GZMK^+^ CD8^+^ T cell accumulation associated with fibroblast-mediated neutrophil chemoattractant production, further demonstrating that inflammatory cell communities in CRSwNP occupy spatially defined niches with distinct cell-cell communication programs (50). The convergence of multiple spatial transcriptomic datasets now makes it possible to map candidate TLS regions within nasal polyp tissue with cellular precision (**Figure 3**).

### 4.2 Depth-dependent TLS phenotypes (working hypothesis)

Based on the available spatial multi-omics data for CRSwNP combined with TLS maturation biology, we propose as a working hypothesis that anatomical location within the nasal mucosa correlates with TLS functional phenotype (**Figure 3**), a principle that, if validated, would have profound implications for both surgical planning and therapeutic targeting. We stress that this model is inferred rather than directly demonstrated. As detailed in Section 4.3, no study has yet co-registered TLS maturation stage with mucosal depth in CRSwNP at single-structure resolution.

Superficial TLS located immediately beneath the surface epithelium (Tier 1-2, corresponding to the lamina propria) are characterized by close spatial coupling to epithelial-derived TSLP and IL-33 signals, high expression of ILC2-activating ligands, and predominantly effector immune profiles (12, 32). These superficial TLS are likely the primary source of ongoing eosinophil and mast cell recruitment during active disease and are accessible to both topical corticosteroids and the diffusion gradients of systemically administered biologics.

By contrast, deep submucosal TLS, particularly those associated with perivascular niches in the periosteal region, exhibit mature GC morphology (Tier 3), enrichment of TCF1^+^ progenitor CD4^+^ TRM cells, reduced TSLP receptor expression, and relative metabolic quiescence (39, 41, 42). Under this hypothesis, these deep TLS would be relatively insulated from epithelial alarm signals and from the pharmacokinetic reach of most circulating monoclonal antibodies. They may therefore represent a durable anatomical substrate of disease memory and a potential driver of polyp recurrence following either surgery or biologic withdrawal, although this proposal awaits direct validation.

This depth-heterogeneity model predicts that standard FESS, which removes surface and lamina propria tissue but preserves the periosteal plane, would be expected to eliminate Tier 1–2 TLS while leaving Tier 3 deep TLS largely intact. Conversely, a Reboot surgical approach targeting the periosteal plane would address both TLS tiers simultaneously, potentially achieving a more complete immunological reset. However, rigorous prospective data validating TLS tier-specific surgical clearance and its correlation with long-term recurrence remain a critical research priority.

### 4.3 Technical limitations of current TLS spatial research in CRSwNP

Scientific integrity demands acknowledgment of the current methodological constraints on TLS spatial research in CRSwNP. First, the spatial resolution of standard 10x Visium (~55 μm spot diameter) remains insufficient to resolve the microarchitecture of individual TLS, which may span only 200-400 μm in diameter within nasal polyp tissue. Visium HD partially addresses this limitation (47, 60). Second, nasal mucosal tissue presents specific processing challenges for spatial transcriptomics, including mucin contamination of cryosections and high RNA degradation risk in submucosal compartments (32). Third, quantitative TLS density standards applicable to CRSwNP, analogous to the Immunoscore or TLS density scoring systems developed in oncology (22, 40), have not yet been established or validated in large prospective rhinological cohorts. Finally, while single-cell transcriptomic atlases of CRSwNP are now highly informative (31, 61), direct spatial mapping of TLS maturation stages specifically in CRSwNP tissue, with confirmation of functional GC reactions by Bcl-6/CD21 immunohistochemistry, remains to be accomplished in adequately powered multicenter studies.

## 5 Clinical translation: the ENT surgeon’s and allergist’s perspective

### 5.1 Redefining surgical boundaries: TLS distribution determines resection depth

The Trifunctional TLS Model demands a fundamental reconceptualization of the therapeutic goals of sinus surgery. The traditional FESS paradigm defines surgical success as restoration of sinonasal patency and macroscopic polyp clearance, with resection planes dictated by anatomical landmarks rather than immunological boundaries (1, 10). TLS biology reframes this as insufficient, suggesting that the true objective may need to shift toward immunological microenvironment reset rather than morphological tissue clearance alone.

TLS biology raises three critical surgical questions. First, does TLS density in CRSwNP tissue vary across anatomical subsites, specifically among the ethmoid mucosa, middle meatal mucosa, and skull base mucosa, in patterns that would mandate subsite-specific surgical extent decisions? Published spatial transcriptomic data suggest heterogeneity in immune density across these regions, though TLS-specific anatomical mapping remains incomplete (31, 32). Second, does the maturation tier of TLS within a given patient’s biopsy predict the likelihood of post-surgical recurrence, analogous to TLS density predicting immunotherapy response in solid tumors (21, 40, 62)? Third, does the radical mucosectomy approach of Reboot surgery achieve superior long-term recurrence control compared with standard FESS (48, 49)?

Existing retrospective data document higher rates of long-term disease control with expanded surgical approaches in severe CRSwNP (9). However, these studies were designed around polyp score and CT score outcomes rather than TLS-specific immune endpoints, and they precede current hypotheses regarding TLS as a potential structural determinant of recurrence. Prospective trials with integrated TLS pathological assessment are urgently needed to validate the TLS-based surgical rationale and to delineate the patient subgroups most likely to benefit from extended resection. Equally important is a balanced appraisal of the risks inherent to radical approaches, including potential olfactory nerve injury, susceptibility of exposed bone to osteitis, and impaired mucociliary regeneration, considerations that temper enthusiasm for maximal surgical extent in the absence of high-quality prospective evidence (1, 9). Given the absence of any prospective data linking TLS clearance to recurrence outcomes, extended or radical mucosectomy should at present be regarded as a hypothesis-driven investigational strategy, not a clinical recommendation.

### 5.2 Biologic limitations and TLS-targeting strategies: beyond the cytokine blockade model

All three currently FDA-approved biologics for CRSwNP, namely dupilumab, mepolizumab, and omalizumab, share a fundamental design logic in which peripheral cytokine or receptor blockade disrupting the systemic type 2 inflammatory cascade to suppress local polyp growth (**Table 2**) (2–5, 7). The TLS Sanctuary Effect model exposes a critical structural weakness in this approach. By operating at the level of circulating mediators, these agents do not address the self-sustaining TLS-resident immune circuits that persist in submucosal compartments (35, 43, 44).

**Table 2:** Approved biologics for CRSwNP and their theoretical interactions with TLS biology.

Biologic	Target	NPS reduction (vs placebo)	Mechanism against TLS tier 1–2	Mechanism against TLS tier 3	TLS sanctuary effect risk	Post-discontinuation rebound
Dupilumab	IL-4Rα	−2.06 (SINUS-24); −1.80 (SINUS-52)	Blocks IL-4/IL-13-driven B cell IgE CSR; reduces Th2 cell generation	Limited: poor GC penetration; low IL-4Rα on quiescent TRM	High	High (6–12 months)
Mepolizumab	IL-5	–0.73 (SYNAPSE)	Reduces eosinophil recruitment to effector TLS zones	Minimal: does not target B cell or T cell components	Moderate–High	High
Omalizumab	IgE	−1.14 (POLYP 1); −0.59 (POLYP 2)	Neutralizes circulating IgE, reducing mast cell/basophil activation	Minimal: does not target TLS structure or T cell memory	High	High
Anti-CXCL13*	CXCL13	Investigational	Disrupts B cell and Tfh chemotaxis into forming TLS	Targets both superficial and deep TLS; theoretically dismantles GC chemokine gradients	Potentially Low	TBD
BTK inhibitors*	BTK (B cell BCR signaling)	Investigational	Impairs TLS B cell activation and IgE production	Targets GC-B cells in deep TLS; complementary to anti-cytokine approach	Potentially Low–Moderate	TBD

*Investigational agents not yet approved for CRSwNP. NPS: nasal polyp score; CSR: class-switch recombination; GC: germinal center; BCR: B cell receptor; BTK: Bruton’s tyrosine kinase; TBD: to be determined.

The columns “Mechanism against TLS Tier 1–2,” “Mechanism against TLS Tier 3,” “TLS Sanctuary Effect Risk,” and “Post-discontinuation Rebound” represent proposed, hypothesis-generating attributions inferred from TLS maturation biology and cross-disease (oncology/immunology) pharmacokinetic data. With the exception of the NPS reduction values (derived from the cited randomized trials), the interactions between these biologics and TLS compartments have *not been directly measured in CRSwNP* and must not be interpreted as established mechanisms or as guidance for clinical agent selection.

Three interconnected questions define the frontier of TLS-biologic interaction research. First, can approved biologics achieve adequate tissue concentrations within mature TLS central zones to modulate resident cellular programs? The dense FDC-stromal network and reduced vascular permeability of mature GCs create pharmacokinetic barriers that may limit drug distribution to the GC center, a limitation well-documented in lymphoma pharmacology and potentially applicable to mucosal TLS (17, 46). Second, are the TLS-resident TRM and B cell populations intrinsically resistant to current biologics? Evidence from nasal polyp T cell phenotyping studies demonstrates that the CD69^+^ CD103^–^ CD4^+^ TRM subset, which shows a Th2-skewed phenotype and is associated with severe disease, expresses markers of low metabolic activity inconsistent with maximal IL-4Rα surface expression (41, 42). Third, can novel agents specifically targeting TLS formation be developed?

Several molecular targets merit evaluation as TLS-directed therapies in CRSwNP. Anti-CXCL13 monoclonal antibodies, by disrupting the principal B cell and Tfh chemokine gradient sustaining TLS integrity, could theoretically destabilize both Tier 2 and Tier 3 TLS (20, 22). Agents targeting LTα1β2 or LTβR signaling would block the core organogenesis pathway of TLS neogenesis and HEV maintenance (17, 46). Bruton’s tyrosine kinase (BTK) inhibitors, by targeting B cell receptor signaling within TLS-resident B cells, could impair GC reactions and local IgE production without requiring direct TLS penetration (39, 63). Each of these strategies would complement rather than replace existing biologic therapies, suggesting that combination approaches targeting both the peripheral cytokine milieu and the TLS structural apparatus may be required to achieve genuine disease modification in severe CRSwNP.

### 5.3 TLS as a prognostic biomarker: from pathology report to individualized treatment decision

The prognostic value of TLS density is now well-established in oncology, where the Immunoscore and TLS-based signatures reliably predict both survival and immunotherapy response across multiple tumor types (21, 22, 40, 62). We propose that an analogous framework is scientifically justified and merits development for future clinical application in CRSwNP (**Table 3**).

**Table 3:** Proposed research TLS grading framework for CRSwNP (not validated for clinical use).

Parameter	Assessment method	Grade 0	Grade 1	Grade 2	Grade 3
CXCL13^+^ B cell aggregates	IHC (semi-quantitative)	Absent	1–2 per 5 HPF	3–5 per 5 HPF	>5 per 5 HPF
Bcl-6^+^ GC structures	IHC (semi-quantitative)	Absent	1 per 5 HPF	2–3 per 5 HPF	>3 per 5 HPF
CD21^+^ FDC networks	IHC (binary)	Absent	Present	Present	Present
PNAd^+^ HEVs	IHC (semi-quantitative)	Absent	Rare	Moderate	Abundant
Composite TLS Score	Sum of above	0 (TLS Absent)	1–4 (TLS Low)	5–8 (TLS Intermediate)	9–12 (TLS High)
Hypothesized biologic response (to be tested)		Excellent (durable)	Good	Partial	Limited (consider surgery first)
Hypothesized recurrence risk (to be tested)		Low	Moderate	High	Very High

HPF, high-power field; IHC, immunohistochemistry; FDC, follicular dendritic cell; HEV, high endothelial venule; GC, germinal center. Cutoffs proposed based on analogy with oncological TLS scoring; prospective validation required.

These cutoffs are extrapolated by analogy from oncological TLS scoring and have not been derived or validated in any retrospective or prospective CRSwNP cohort. This framework is intended solely to structure future validation studies and must not be used to guide clinical decisions, including decisions regarding extended or radical surgery.

**Figure 4:**
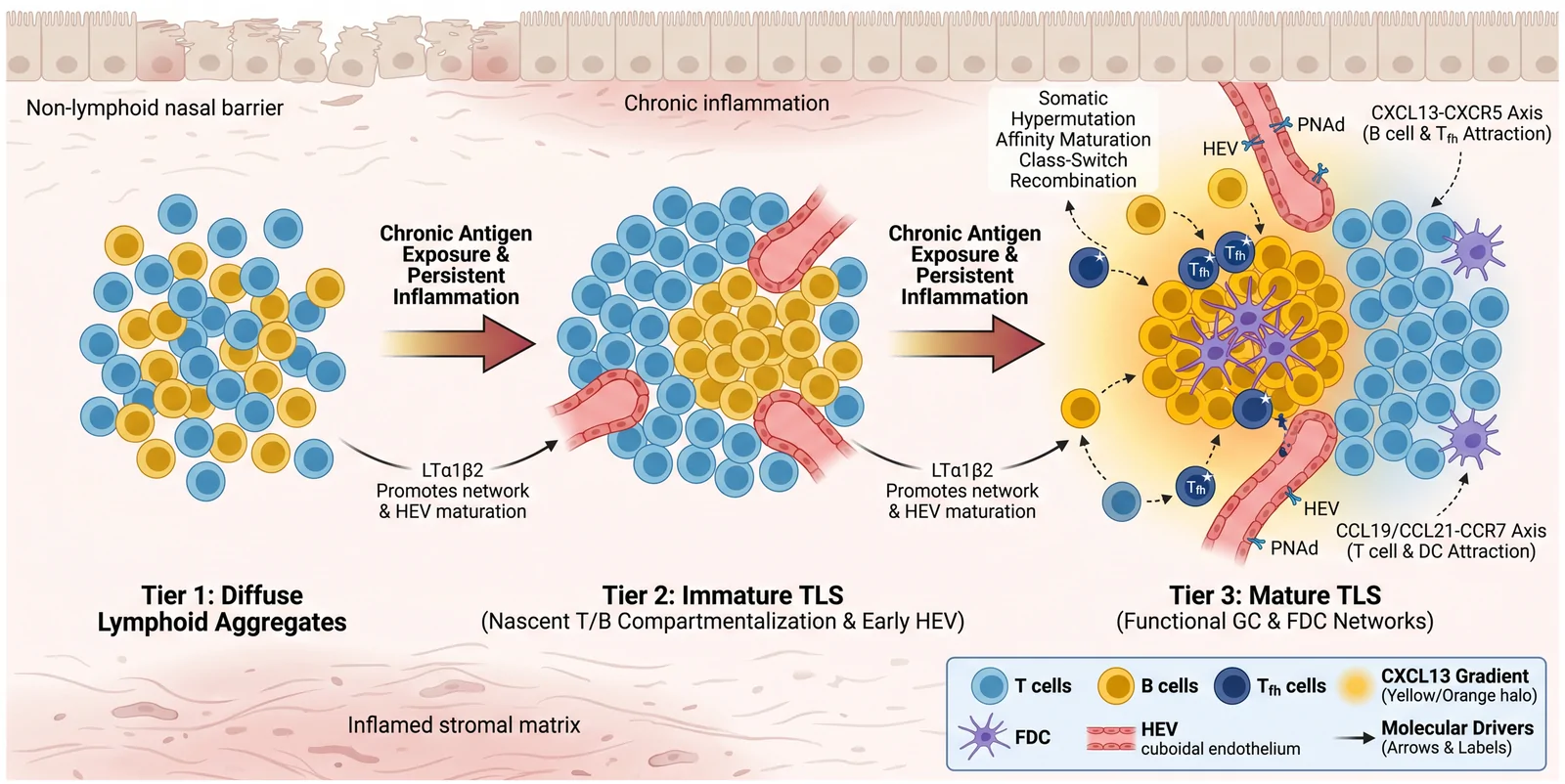
Proposed research algorithm illustrating how a TLS grading framework could, in principle, be integrated into CRSwNP treatment decision-making. This algorithm is a hypothesis-generating construct intended to structure future validation studies and is not validated for clinical use and must not be used to guide clinical decisions. Pathological specimens from FESS are assessed for CXCL13+ B cell aggregate density (early TLS marker) and Bcl-6+/CD21+ GC structures (mature TLS marker) using semi-quantitative immunohistochemistry. Patients are stratified into TLS Low (Tier 0-1), TLS Intermediate (Tier 1-2), and TLS High (Tier 2-3) categories. TLS Low patients are hypothesized to be candidates for biologic monotherapy with anticipated durable response. TLS Intermediate patients may be candidates for early combination biologic and surgical therapy. TLS High patients would be flagged for discussion of extended resection approaches followed by biologic therapy, with close recurrence surveillance. All treatment associations shown are hypothesized rather than evidence-based, and prospective validation of this algorithm is required before any clinical application.

Specifically, we propose, as a hypothesis for future validation, that pathological assessment protocols in CRSwNP research should evaluate, alongside standard eosinophil density and mucosal remodeling parameters, semi-quantitative scoring of two TLS-defining immunohistochemical markers, namely CXCL13^+^ B cell aggregates (as an early/intermediate TLS indicator) and Bcl-6^+^/CD21^+^ GC structures (as a mature TLS indicator). A TLS High pathological profile, defined provisionally as more than 3 Bcl-6^+/^CD21^+^ GC structures per high-power field across a minimum of 5 tissue sections, may identify patients at high risk of biologic monotherapy failure and early surgical recurrence who may therefore benefit most from early combined surgical and biologic interventions. Conversely, patients with TLS Low profiles may represent candidates for biologic monotherapy with anticipated durable response and reduced recurrence risk (**Figure 4**).

This TLS-stratified precision medicine approach parallels the logic of eosinophil count-guided biologic selection that has been validated in asthma and CRSwNP management (64, 65). A key advantage of the proposed TLS grading framework is that it directly reflects the structural immunological architecture determining therapeutic vulnerability, rather than serving as a surrogate biomarker of an underlying molecular state. The network meta-analysis of biologics for CRSwNP demonstrating differential outcomes across patient subgroups (66) provides additional motivation for developing biomarker frameworks that move beyond aggregate population-level efficacy data toward individually tailored treatment selection.

## 6 Future perspectives: three fundable scientific questions

The research directions generated by the Trifunctional TLS Model are specific, mechanistically grounded, and amenable to near-term investigation. We propose three priority scientific questions that represent the most impactful targets for future research funding and clinical trial design.

Question 1. Does early biological intervention in CRSwNP prevent the structural maturation of TLS from diffuse lymphoid aggregates to mature GC-containing structures, thereby achieving true disease modification?

The temporal dimension of TLS formation, requiring sustained chronic inflammation over years, suggests a window of opportunity for early intervention. A prospective randomized trial comparing TLS density and maturation tier in biopsies obtained at 6 and 24 months following early dupilumab initiation (at first diagnosis) versus standard stepwise therapy would directly test this hypothesis. If early biologic initiation forestalls TLS maturation in a subset of patients, this would constitute the first mechanistic evidence for disease modification in CRSwNP, fundamentally reshaping clinical guidelines currently recommending biologic therapy only after repeated surgical failure (1, 6, 12).

Question 2: Is there a causal, bidirectional relationship between specific S. aureus virulence factors and TLS density in CRSwNP, and do mature TLS provide a protected ecological niche that shields S. aureus from mucosal immune clearance?

The superantigen-TLS axis identified in this review suggests a self-reinforcing cycle in which *S. aureus* superantigens drive TLS formation, while TLS-resident plasma cells generate IgE rather than bactericidal IgG against *S. aureus* antigens, impairing microbial clearance and enabling persistent colonization (24, 25, 27, 67). Mechanistic validation of this cycle using spatially resolved metagenomics combined with TLS density mapping in CRSwNP specimens would open a novel therapeutic avenue, namely targeting sinonasal *S. aureus* colonization (through topical bacteriophage therapy, anti-biofilm agents, or microbiome remodeling strategies) to indirectly dismantle TLS and prevent their reformation (9, 26).

Question 3: What is the quantitative impact of repeated sinus surgery, particularly Reboot-style radical mucosectomy, on TLS density and maturation tier, and does surgically achieved TLS reduction synergize with subsequent biologic therapy to produce superior long-term outcomes compared to either modality alone?

The question of optimal sequencing of surgical and biologic interventions remains among the most pressing unresolved issues in CRSwNP management (11, 68). A prospective cohort study incorporating paired pre- and post-surgical TLS-specific immunohistochemistry, followed by randomization to biologic versus observation arms, would provide the first direct evidence for or against a surgically driven immune reset capable of enhancing the durability of subsequent biologic responses. This design would also enable identification of the TLS-density threshold below which biologic therapy alone achieves long-term remission, directly informing the sequential versus combination treatment debate.

## 7 Conclusions

The proposed Trifunctional TLS Model offers a candidate structural-immunological framework that may help reconcile three enduring paradoxes of CRSwNP, namely local IgE synthesis without systemic sensitization, high polyp recurrence after complete surgical clearance, and disease rebound after cessation of otherwise effective biologic therapy. Within this framework, mature TLS are hypothesized to act as local IgE-producing factories, as protective niches for disease-sustaining TRM cells that may be relatively refractory to systemic biologics, and as semi-autonomous immune centers that could contribute to polyp recurrence independently of systemic signals. Dissection of the likely cellular constituents, including GZMK^+^ CD8^+^ T cells, GC-B and IgE-switched plasmablast populations, atypical/age-associated B cells, and a Tfh13-amplified, Tfr-restrained follicular axis, converts these functional labels into specific, testable cellular hypotheses. Spatial transcriptomic data raise, but do not yet demonstrate, the possibility that TLS anatomical depth influences functional phenotype and therapeutic vulnerability. This depth-dependent model remains a working hypothesis pending single-structure co-registration of maturation stage with mucosal depth.

Crucially, this framework applies most plausibly to severe, type 2-high CRSwNP and must be interpreted against the disease’s endotypic and geographic heterogeneity and against well-established TLS-independent mechanisms, including epithelial barrier dysfunction, basal-cell reprogramming, and innate lymphoid memory, that may operate in parallel. The proposed TLS grading framework is offered strictly as a research instrument to structure future validation studies and is not validated for clinical use and must not be used to guide clinical decisions. Looking forward, the three research questions posed here, namely early intervention to prevent TLS maturation, the *S. aureus and* TLS feedback loop, and surgical TLS clearance as a platform for biologic synergy, define a translational agenda situating CRSwNP TLS biology at the interface of structural immunology, precision otolaryngology, and next-generation biologic design. If these hypotheses withstand rigorous prospective testing, the field may ultimately move beyond a purely suppressive paradigm toward interventions capable of genuine immunological restructuring of the nasal mucosa.
